# Supplementary material for: Altitudinal range-size distribution of breeding birds and environmental factors for the determination of species richness: An empirical test of altitudinal Rapoport’s rule and non-directional rescue effect on a local scale
Source: PLoS One. 2019 Jan 25;14(1):e0203511. doi: 10.1371/journal.pone.0203511 (PMC6347176; doi:10.1371/journal.pone.0203511)
Supplement: S1 Table — Bold = correlated predictor (r ≥ |0.7|). (PDF) [file pone.0203511.s001.pdf]

S1 Table. Pearson's correlations between climatic (maximum temperature and minimum temperature), vertical (coverage of understory, midstory, and overstory vegetation) and horizontal habitat heterogeneity (habitat diversity). Bold = correlated predictor (r ≥ |0.7|).

	Maximum temperature	Minimum temperature	Understory vegetation	Midstory vegetation	Oversotry vegetation	Habitat diversity
Maximum temperature	1					
Minimum temperature	**0.991**	1				
Understory vegetation	-0.326	-0.304	1			
Midstory vegetation	0.117	0.133	0.053	1		
Oversotry vegetation	-0.184	-0.131	-0.039	-0.103	1	
Habitat diversity	0.665	0.659	-0.089	0.156	-0.243	1
