# Supplementary material for: Altitudinal range-size distribution of breeding birds and environmental factors for the determination of species richness: An empirical test of altitudinal Rapoport’s rule and non-directional rescue effect on a local scale
Source: PLoS One. 2019 Jan 25;14(1):e0203511. doi: 10.1371/journal.pone.0203511 (PMC6347176; doi:10.1371/journal.pone.0203511)
Supplement: S2 Table — (PDF) [file pone.0203511.s002.pdf]

S2 Table. Information of the study plots along altitudinal gradient.

Range of elevation (m)	Range of latitude (°N)	Range of longitude (°E)	No. of plots
200-300	35°23'46.3"~35°15'09.3"	127°49'56.2"~127°28'02.9"	10
300-400	35°24'42.5"~35°16'07.1"	127°48'59.7"~127°28'03.6"	11
400-500	35°23'45.7"~35°16'04.7"	127°47'56.9"~127°29'03.3"	13
500-600	35°21'58.8"~35°17'01.9"	127°47'57.2"~127°29'02.5"	12
600-700	35°22'42.2"~35°17'04.6"	127°46'59.7"~127°29'03.5"	12
700-800	35°22'46.6"~35°17'08.2"	127°46'39.1"~127°29'00.5"	12
800-900	35°22'57.3"~35°18'00.1"	127°46'57.6"~127°29'04.0"	12
900-1000	35°21'50.4"~35°18'01.1"	127°45'56.2"~127°29'00.3"	12
1000-1100	35°21'37.9"~35°19'05.3"	127°45'56.9"~127°30'03.8"	12
1100-1200	35°20'59.1"~35°18'08.9"	127°45'54.3"~127°30'02.0"	12
1200-1300	35°20'50.8"~35°17'28.3"	127°45'38.4"~127°31'02.0"	12
1300-1400	35°20'51.2"~35°17'31.6"	127°45'34.8"~127°31'04.8"	12
