# Supplementary material for: Altitudinal range-size distribution of breeding birds and environmental factors for the determination of species richness: An empirical test of altitudinal Rapoport’s rule and non-directional rescue effect on a local scale
Source: PLoS One. 2019 Jan 25;14(1):e0203511. doi: 10.1371/journal.pone.0203511 (PMC6347176; doi:10.1371/journal.pone.0203511)
Supplement: S3 Table — (PDF) [file pone.0203511.s003.pdf]

S3 Table. Results of the AICc based multimodel inference of species richness of 1st and 4th quartile.

**Parameter**	**Model-averaged estimates**	**SE**	***P*-value**	**Importance value**
**Species richness of 1st quartile**				
Intercept	0.049	0.19	0.801	-
Minimum temperature	0.017	0.04	0.638	0.25
Habitat diversity	0.422	0.09	**< 0.001** ***	1.00
Understory vegetation	-0.053	0.05	0.278	0.10
Midstory vegetation	-0.054	0.05	0.318	0.10
Overstory vegetation	0.022	0.05	0.660	0.10
**Species richness of 4th quartile**				
Intercept	6.445	1.52	**< 0.001** ***	-
Minimum temperature	-0.331	0.12	**0.006** **	0.91
Habitat diversity	-0.476	0.46	0.302	0.38
Understory vegetation	0.154	0.18	0.403	1.00
Midstory vegetation	-0.089	0.19	0.636	1.00
Overstory vegetation	0.818	0.17	**< 0.001** ***	1.00

Candidate models included those with Akaike weight within 10 % of the highest value. Asterisks *** indicate $P < 0.001$; **, $P < 0.01$; *, $P < 0.05$. SE = standard error.
